# Reviews and Book Notices

**Published:** 1881-01

**Authors:** 


### Reviews and Book Notices.

ARTICLE XII.—THE PHYSIOLOGY OF WOMAN; embracing Girlhood, Maternity and Mature Age, with Essays on "Co-education of the Sexes in Medicine," "The Physiological Basis of Education," "Temperance from a Physician's Point of View," and "A Plea for Moderation." By Sarah Hackett Stevenson, M.D., Adjunct Professor of Obstetrics, and late Professor of Physiology in the Woman's Medical College of Chicago. Cushing, Thomas & Co., Chicago; 1880.

This book conveys some information which is sadly needed. In the introduction the author disclaims any intention of having endeavored to write a "*medical book.*" The volume contains no formulas. It is a book of *advice and instruction* to women, couched in the plainest terms and clearest language. No wife or mother, who will read the book carefully and thoughtfully can fail to understand every sentence in it. The information conveyed is also of the most *practical* kind. And besides that, it is of a kind which is needed. The average woman goes through the world in total ignorance of some of the most important facts pertaining to her condition as a wife and mother. Dr. Stevenson has succeeded admirably in selecting the topics which are perhaps of the greatest importance to women in every-day life, and has therefore written a book which is *useful.*

On the 107th page the statement is made that "there are no cases on record of death from *chloroform during labor.*" This assertion needs to be modified somewhat. That there is greater immunity from danger in administering chloroform during labor than in non-parturient cases, the profession will pretty generally admit, but the fact that there are on record authentic reports of death from the inhalation of chloroform during child-birth, should be sufficient to make every physician careful in its use.*

On the 108th page this sentence occurs: "I consider the moderate use of chloroform *perfectly free from danger* in the first and second stages of labor." Were this book intended for the use of practitioners, this statement would be received with due allowance; but the fact that persons, inexperienced in the use of chloroform, will read this book, carries with it an element of danger, in that it does not lay sufficient emphasis upon the fact that the administration of chloroform is not free from danger *under any circumstances.* With this exception the advice given for the care of the parturient woman and new-born child is excellent.

The author touches upon several points not treated by medical writers generally. The question, "Who shall tell the children of their origin?" is answered in a very satisfactory way. The four essays mentioned in the title of this work are able and well written. Taken all in all, this volume is one which deserves a careful reading by every mother in the land. The typography of this work is of an inferior character. The binding is very imperfectly and cheaply done. B. W. G.

ARTICLE XIII.—CONSUMPTION; AND HOW TO PREVENT IT. By Thos. J. Mays, M.D., Member Pennsylvania Medical Society, Author of "On the Therapeutic Forces," etc. New York: G. P. Putnam's Sons.

In the introduction to this book the author portrays most vividly the wide-spread and terrible fatality of consumption. The number of deaths from this disease in the United States, during the year 1870, was 69,896. Here is another startling statement: "The whole of Maine and New Hampshire, the most of Vermont, Massachusetts, and Connecticut, and all of Northern New York, show that *two thousand* (2,000) out of every *ten thousand* (10,000) who die, owe their death to consumption."

Dr. Mays argues that if physicians could only be paid for *preventing disease*, the high mortality of these regions above men-

* See paper by Prof. Wm. T. Lusk, M.D., of New York, on the "Necessity of Caution in the Use of Chloroform during Labor."

tioned, might be lowered. He then, after briefly considering the nature of consumption, proceeds to enumerate the prophylactic agencies that can be brought to bear against this "ghastly enemy of mankind." The influence of foods, air, soils, clothing, light, physical exercises, digestion, disease, infant diet, cod liver oil and other fats, as factors in the causation or prevention of consumption, is affirmed, by the author to be very great.

On page eighty-seven the relation of alcohol to disease is considered. It is called "a double-edged instrument," powerful for good or evil. But there is no doubt that the author states the opinion of the very large majority of medical men when he advises the moderate and guarded use of alcohol in consumption.

The book is written with a spirit of candor and consistency which ought to give it a reading by a great many physicians.

The hygienist has in this book a suggestion which is worthy of consideration. B. W. G.

ARTICLE XIV.—THE ART OF PROLONGING LIFE. By Christopher William Hufeland. Edited by Erasmus Wilson, M.D., Author of "A System of Human Anatomy," etc. From the last London Edition. 12mo., pp. 300. Lindsay & Blakiston, Philadelphia: 1880.

The author, a German philosophic physician, lived a hundred years ago, but his work is very modern on account of his advanced views. The philosophy of the subject is deep, evident, simple and clear; worthy of recent naturalists, as these quotations show:

"I can at any rate assert that physical and moral health are as nearly related as the body and the soul.

"The business of thinking itself, as carried on in this mortal machine, is organic."

It is obvious that medicine had not yet reached its present scientific stand-point, since the author says that "Many diseases may be the means of prolonging life," and "The application of medicine is nothing else than exciting an artificial disease, in order to expel one that is natural."

The editor has ably amended some similar faults, and added very interesting notes, which might be more numerous.

A feature of this book is that it was written for the public as well as for the profession, and is very interesting to both. Every scientific man should have a copy of it on his shelf.

The first part is a sketch of the history of medicine and hygiene, and of comparative life, with valuable statistics. The second part treats of the (well-known) means which shorten life, and the third and last, of the means which prolong life.

H. D. V.

ARTICLE XV.—HEALTH AND HOW TO PROMOTE IT. By Richard McSherry, M.D., Professor of Practice of Medicine, University of Maryland; President of Baltimore Academy of Medicine, etc. D. Appleton & Co., 1879. New York.

Hygiene, both public and private, is a subject of ever increasing importance. When it is better appreciated and understood, therapeutic measures can be more effectively carried out, for then the medical profession will have, to a greater or less extent, the co-operation of the laity. Dr. McSherry has done a good work. He has not attempted to write a book for professional men alone.

The general public will read it with interest for two reasons: First, it contains a vast deal of information in which the reading public is deeply concerned. Second, it is written in a style which is free from technicalities, and which pleases while it instructs. Part II is especially entertaining, and at the same time eminently practical. We commend the book to those who are interested in preventive medicine. B. W. G.

ARTICLE XVI.—CLINICAL LECTURES ON DISEASES PECULIAR TO WOMEN. By Lombe Atthill, M.D., Master of the Rotunda Hospital, Dublin, etc., etc. Fifth edition. Illustrated. Philadelphia: Lindsay & Blakiston; 1879.

Like most other books, this volume has its faults and its excellencies. The style is clear and perspicuous. There is no ambiguity about it. Dr. Atthill has the enviable faculty of saying what he wants to say, without any circumlocution. But the manner is perhaps, better than the matter. There is nothing new in this book. In fact there are some things advocated and recommended which are not abreast of the age. Fergusson's speculum is recommended for "*general use.*" It is certain that there are several patterns of bi-valve specula infinitely to be preferred to Fergusson's for "*general use.*" Again Dr. Atthill says "some practitioners have a great dread of applying caustics to the interior of the uterus, *a fear which is totally groundless.*"

This is plainly unsound and unsafe doctrine. In well selected cases this practice may be followed, but that there is need of caution in the employment of nitric acid and similar agents, is admitted by the very large majority of practitioners in this country. The author's teachings in these lectures are calculated to give a feeling of unconcern and almost recklessness in making topical applications to the endometrium.

Want of space prevents a detailed notice of all these lectures. Taken as a whole, the book is a good one to read. It is written by a man of large experience in his profession. B. W. G.

